# Attitudes toward vaccines during the COVID-19 pandemic: results from HBCU students

**DOI:** 10.3934/publichealth.2022012

**Published:** 2021-12-17

**Authors:** Richard Moye, Antonius Skipper, Tangela Towns, Daniel Rose

**Affiliations:** 1 Department of Behavioral Sciences, Winston Salem State University, Winston Salem, NC, USA; 2 Gerontology Institute, Georgia State University, Atlanta, GA, USA

**Keywords:** vaccine hesitancy, COVID-19, coronavirus, college students, pandemic, online survey, mixed methods, public health communication, racial disparities, HBCU

## Abstract

**Purpose:**

To investigate the prevalence of vaccine hesitancy among black college students and to explain students' reasoning behind their vaccine hesitancy.

**Design:**

online survey completed in spring and summer of 2021. Students were recruited via email.

**Setting:**

HBCU campus, North Carolina, USA.

**Subjects:**

397 currently enrolled students.

**Measures:**

An original survey instrument was developed which included questions on vaccination status and plans to get vaccinated, perceived threat from the coronavirus (adopted from PEW research) exercise behaviors and demographics. Respondents were also given the chance to respond to an open-ended question about their feelings about the vaccine.

**Analysis:**

Binary Logistic Regression predicting likelihood that respondent is vaccine hesitant.

**Results:**

Confidence in the safety of the vaccine was the strongest predictor of vaccine hesitancy. At the time of the survey only 25% of students had received at least one dose of the vaccine. 37% of the students did not plan on ever getting vaccinated. Other considerations (living with a vulnerable person or concerns about their own vulnerability to COVID) were not associated with getting vaccinated. Students were particularly concerned about side effects from the vaccine.

**Conclusion:**

Racial disparities in COVID-19 infections, deaths, and vaccinations serve as a stark reminder of the urgent need to better understand the factors that could lead to mitigation of the virus. Fear about the safety of the vaccine among minority populations in particular must be unpacked in order to address valid concerns and overcome hesitancy. This study provides key insights into the contours of those fears.

## 1. Introduction

As of June 7, 2021, 1 in 470 African Americans have died as a result of COVID-19 [1]. The Centers for Disease Control (CDC) estimates the COVID-19 death rates for African Americans to be twice as high as White, non-Hispanic persons [2]. Despite the rollout of a COVID-19 vaccine, COVID-related death rates remain high for African Americans as a result of, at least in part, to the institutional and structural racism and its effects on health and health outcomes [3],[4]. The consequence of historical events grounded in racism has led to vaccine hesitancy of many African Americans [5]. The World Health Organization (WHO) describes vaccine hesitancy as the “delay in acceptance or refusal of vaccines despite availability of vaccine services [6]. Research broadly attributes this delay or refusal to confidence, complacency, and/or convenience, where confidence represents trust and safety, complacency represents perceived risk, and convenience represents access [7]. In this vein, the disproportionate effect of COVID-19 on minority populations, and particularly African Americans, has led to several efforts to recognize and overcome vaccine hesitancy.

Multiple researchers note the historical and contemporaneous institutional distrust associated with vaccination decisions as a mechanism driving vaccine hesitancy among African Americans [5],[8]. Whether medical, public health, or governmental, African Americans have long experienced persistent racism and inadequate care in the U.S. [8]. Past transgressions such as the Tuskegee Syphilis Experiment lead to residual effects in which African Americans may adopt views that counter experts and contribute to decreased adherence to medical advice [9],[10]. However, given the recent emergence and effects of COVID-19, the mechanisms surrounding vaccine hesitancy among African Americans, particularly during young adulthood, are less understood.

### 1.1. Science behind the vaccine

Vaccine hesitancy might be influenced by the general public's lack of understanding in virology, vaccine development, and the research development process. Many in the United States enjoy benefits of public health interventions and the historically lengthy process of vaccine development, such as extended life expectancy and quality of life. This is illustrated by CDC 2019 data suggesting that 92.6% of children had received the Polio vaccination by 24 months of age [11]. Amid questions about perceived susceptibility, extreme severity of disease, increased health barriers, and culturally sensitive barriers, questions about the efficacy of COVID-19 vaccination remain due to lack of confidence in (or understanding of) the vaccine.

Although COVID-19 (SARS-CoV-2) is a novel virus; the Coronaviruses (CoVs), Severe Acute Respiratory Syndrome (SARS) viruses, and MERs-CoV are groups of respiratory viruses that have been studied extensively and thus researchers understand the biological characteristics of these viruses well enough for foundational knowledge and vaccine development [12],[13]. Scientists used this basic understanding in their exploration of the novel COVID-19 (SARS-CoV-2), its symptoms, transmission, and viral function [13].

In response to the global threat of the COVID-19's spread, haste was made to improve upon global public health as the pandemic began. The etiology of the COVID-19 virus was discovered late 2019 [12]. Governmental protocols and FDA oversight to ensure safety, effectiveness, and quality of products warrants the virus be researched and tested in the laboratory [14]. Preclinical research is then conducted in the laboratory ensuring the vaccines are sterile, pure, potent, and consistent across their mass production [14]. After approval of the preclinical research, clinical trials with volunteers begin. This process withstands 3 trial phases including participants in the thousands and comparative measures prior to the manufacturing process [14]. Though numerous clinical trials, strategies, and types of vaccines were attempted globally (i.e. protein subunit vaccines, virus-like particle vaccines, DNA vaccines, viral vector vaccines, etc.), many were unsuccessful due to the mechanism and nature of the COVID-19 virus [13]. It is important to note that prior to 2020 there were no effective vaccinations approved for the prevention of any CoV [13]. The literature suggests RNA vaccines and Protein Subunit vaccines as successful [13]. In the United States, the two leading vaccine types are Moderna's & Pfizer's mRNA vaccines along with the Johnson & Johnson's viral vector vaccine [15].

While previous research has addressed the concept of vaccine hesitancy in general, usually towards the polio, measles, mumps, and rubella vaccines typically administered to children or to HPV administered to teenagers, researchers are still in the very early stages of understanding people's attitudes toward the vaccines developed specifically to address COVID-19. Given the novelty of vaccine hesitancy and COVID-19, this next section of the literature review will cover findings from peer reviewed research where researchers gathered data from people during the 2020 pandemic and asked respondents about the topic of vaccine hesitancy as it relates specifically to a vaccine for COVID-19. Nearly all of this research was conducted while the vaccine was being developed and remained in early stages of trials, well before its emergency use authorization and even further before the vaccine was produced and distributed widely enough for most adults to be eligible for vaccination in the United States.

### 1.2. Social response and vaccine hesitancy: a culturally diverse perspective

Cascini et al. (2021) [16] published a systematic analysis of global research on attitudes toward the vaccine and vaccine hesitancy. The analysis included 209 peer-reviewed research studies that had been published prior to July 5, 2021. The majority of the studies were conducted outside of the US, primarily in Asia (n = 77) and Europe (n = 53), but the review also included studies from the general population of the United States, including one focused on college students [17] and one focused on young people 14–24, an age group which includes college students [18]. Patil et al. (2021) [17] found only 48% of young people were willing to be vaccinated, while the Brandt study found 76% of college students were willing to be vaccinated. However, both collected data during 2020, well before the vaccine was available. Cascini et al. (2021) [16] also found that in the U.S. longitudinal studies, people's willingness to vaccinate was decreasing over time as the vaccine became more of a reality and less of an abstract idea. Another study conducted early in the pandemic which focused on the general population of the U.S. provides evidence for racial differences, finding that black adults were less willing to receive a vaccine than white adults [19].

Some of the previous research has pointed to fear of COVID as a strong predictor of intentions to get vaccinated [20],[21]. Karlsson et al. (2021) [20] conducted a multi-study that included 825 Finnish parents, 205 individuals in a suboptimal vaccination coverage region, and 1325 online respondents. The researchers concluded that vaccine hesitancy was most influenced by fears related to the perceived safety of the COVID-19 vaccine. Similarly, Gagneaux-Bronon et al. (2021) [21] collected data from healthcare workers in France and found that vaccine hesitancy was a barrier to receiving the COVID-19 vaccine.

In contrast to the work of Karlsson et al. (2021) [20] and Gagneaux-Bronson et al. (2021) [21], Krepps et al. (2021) [22] found in a study of 1096 Americans that high levels of vaccine efficacy would predict high rates of vaccine acceptance. The role of vaccine efficacy is further shared in the work of Kaplan and Milstein (2021) [23], in which they reported that vaccine efficacy significantly impacts vaccination acceptance. Specifically, the researchers U.S.ed an experimental design to test respondents' stated likelihood of getting vaccinated if the efficacy of the vaccine was 50% effective, 70% effective, and 90% effective. The study's respondents did not have concerns over vaccine side effects and would not link side effects to the rate of vaccine acceptance.

Finally, Griffith, Marani, and Monkman (2021) [24] performed a content analysis of tweets from December 2020, with the aim to identify common themes related to vaccine hesitancy. They found concerns over safety and suspicion about the vaccine development process were the two major driving forces behind vaccine hesitancy expressed on social media. With young adults maintaining a significant presence on social media platforms, along with the speed in which COVID-19-related messages and beliefs can be shared through social media [25], understanding aspects of vaccine hesitancy among younger adults is particularly important.

Overall, previous research points to several hypotheses about factors that might influence vaccine hesitancy. These include: (1) people who are concerned about getting sick from COVID-19 will be more likely to get vaccinated, and (2) people who believe the vaccine is effective will be more likely to get vaccinated. While this research has helped us to develop hypotheses to better understand the factors related to vaccine hesitancy, it is important to go further and observe how attitudes and behaviors differ once the vaccine is available. Further, it is of particular importance to understand vaccine hesitancy among groups that are underserved and underrepresented in clinical research [26]. With that, the present study places emphasis on students at Historically Black Colleges and Universities (HBCUs).

### 1.3. Intersectionality: African American college students and COVID-19

HBCUs and their students have faced additional challenges compared to other institutions during the COVID-19 pandemic. Murty and Payne (2021) [27] discuss the “parallel pandemics” of COVID-19 and racism that have put African American students at higher risk of financial challenges, mental health issues, loss of family members, and lack of access to health care, among other racialized structural barriers. At the institutional level, HBCUs have faced funding gaps, accreditation challenges, and other disruptions to the provision of their important roles [27]. Despite pleadings from two notable HBCU presidents, only 1–2 percent of students at their universities answered the call to participate in vaccine trials [28].

Batelaan (2021) [29] has called for reframing vaccine skepticism in Black communities as “anti-scientist” instead of “anti-science” due to endemic racism in medical communities and structural inequalities in health care. Other researchers have linked barriers African Americans have faced accessing testing, treatment, and vaccines for COVID-19 to acceptance rates of the current vaccines [30]. They further call upon minority pharmacists (who are underrepresented in the field) to help build minority trust in the vaccines.

Synthesizing this literature, the importance of African American medical professionals in building trust of the vaccine has been undermined by centuries of exclusion from the health sciences. These barriers remain in place at the training grounds for many future Black medical professionals, even HBCUs, where Black STEM students report being referred to as “under-intelligent” by non-Black faculty, doctoral students, and postdoctoral researchers [31]. All factors considered, understanding vaccine hesitancy among individuals within HBCUs is timely and important in efforts to overcome barriers to vaccination during and after the COVID-19 pandemic.

### 1.4. Guiding framework

The present study is guided by existing research on the Health Belief Model (HBM). The HBM is led by the belief that actions are informed by the perceived effectiveness of health-related behaviors [32]. More specifically, and in relation to COVID-19, the framework of HBM would assume that beliefs surrounding susceptibility, severity, benefits, barriers, and health motivation would be associated with preventive decisions and vaccine hesitancy. This assumption aligns with findings that suggest beliefs are the leading personal contributor to refusing vaccination [33]. The HBM also notes that cues to action can affect health-related decisions [34]. While the cues to action surrounding the COVID-19 vaccine may include vaccination campaigns and incentives, such efforts remain underexplored when juxtaposed with the many vaccination barriers present in the African American community. Therefore, understanding the perceptions of African American young adults is essential to developing culturally-sensitive cues that can effectively address vaccine hesitancy.

## 2. Methods and data

We developed an online survey to collect data from students on their behaviors and attitudes toward the vaccine. Additionally, we asked about their living situations and other behaviors, such as adherence to social distancing norms during the pandemic.

Students completed the survey over two different time periods. Some students completed the survey during the end of the spring 2021 semester, when the vaccine was available to the older age group (65+) and to healthcare professionals but had not yet been made available to the general public. A handful of students might have been eligible earlier due to being employed in healthcare-related occupations, but others would not have been eligible to receive the vaccine until April 7^th^. April 7^th^ is the date when the vaccine became available to anyone over the age of 16 in the state of North Carolina [35]. Additionally, throughout April, there were only limited opportunities for appointments to receive the vaccine.

A second round of invitations to the survey were distributed during mid-June of 2021 when the vaccine had been made available to all adults and when public health efforts were focused on convincing everyone to take the vaccine. In total, 397 students completed the survey. Approximately 28% were freshman, and 24% sophomores, 24% juniors and 22% seniors. The median age was 20, and the mean age was 22.7. Thirteen percent of the respondents identified their gender as male and 86% identified as female. Forty-two percent of the respondents lived on campus. The key question we asked was: “If the vaccine to prevent COVID-19 were available to you today, would you...” 1) Probably get the vaccine; 2) Definitely get the vaccine; 3) Probably NOT get the vaccine; 4) Definitely NOT get the vaccine or 5) I've already gotten at least one dose of the vaccine.

**Table 1.** Survey attempts by date: March, April, May, June, July.

Attempts by Month		
March	162	41%
April	45	11%
May	10	3%
June	88	22%
July	92	23%
Total	397	100%

Even as the vaccine was being rolled out and made available to all adults across the state, and the University hosted events to get shots in arms on campus, the total number of students who had gotten the vaccine remained low throughout March and April. In fact, across the state there were delays in getting appointments and shipping delays which impacted the availability of the vaccine.

Due to these factors, we have chosen to simplify our analysis by splitting the respondents into two groups based on the date that they participated in the survey. The first group of students participated in March, April, and May. A total of 217 students responded to the survey during this time. Of those, 213 completed the survey. Of those 213, only 25 reported having already received at least one dose of the vaccine at the time that they took the survey.

The second group is students who participated in June and July, when the vaccine was readily available to any adult interested in being vaccinated. By this point in North Carolina, and across the U.S., supply was no longer a problem. Local and state and federal media campaigns were being rolled out to encourage people to become vaccinated, including targeted campaigns at African Americans through radio, TV, and social media. During that time the researchers emailed students enrolled in summer courses. Of the 184 students who responded to the survey during the summer, 71 reported having received at least one dose of the vaccine. While this is a substantial increase from the spring, it is also far lower than the target set by U.S. President Joe Biden for the U.S. to achieve herd immunity (70%).

There were a few differences in the demographics of our sample for those who completed the survey during the spring compared to those who completed the survey during the summer. The students who completed the survey in the spring were generally younger, with a median age of 19 compared to a median age of 21 for the students who completed the survey in the summer. Students who completed the survey during the summer were more likely to be living off campus, as nearly all summer courses were offered online compared to hybrid and face-to-face modalities during the spring semester. These demographic differences between the spring respondents and the summer respondents were not associated with significant differences in respondents' attitudes toward the coronavirus generally: 77% of respondents in the spring reported that the coronavirus was only a minor threat or not a threat to them personally, compared to 81% of respondents in the summer. In addition, 57% of respondents in the spring reported confidence that the vaccine was safe and effective, compared to 62% of respondents in the summer. There was no difference in the percentage of students who reported living with or caregiving for someone over the age of 65 (and thus more vulnerable to the effects of COVID-19).

**Table 2.** Percent vaccinated.

	Number Vaccinated	Total Respondents	Percent of Respondents Vaccinated
March/April/May	25	213	12%
June/July	71	184	39%
Total	96	397	

We asked a series of questions about perceptions of threat from the coronavirus, as well as our key question about plans to get the vaccine. The tables below show the frequency tables for each of those key questions. These questions were adapted from the PEW research survey on COVID vaccine [36].

First, we asked, how much of a threat is the coronavirus outbreak for your personal health? Over half of the respondents described the coronavirus as a minor threat, while about 29% described it as not a threat. Only 20% described the coronavirus as a major threat to their personal health.

We also asked how concerned, if at all, are you that you might spread the coronavirus to other people. 12.6% were not at all concerned, and an additional 24.2% were not too concerned about spreading coronavirus to other people. However, the modal response here is “very concerned”, and the second most selected option was “somewhat concerned”, so respondents might not think that the coronavirus was a big threat to them personally but many of them were generally concerned with spreading it to people around them.

We also asked, “How concerned, if at all, are you that you will get the coronavirus and require hospitalization?” Twenty percent were not at all concerned, and another 39% were not too concerned. Only 17.6% were very concerned about getting a serious case, and 23.4% were somewhat concerned. These percentages reflect slightly lower concern than Americans overall reported in a national survey such as the November 2020 PEW research center survey, where 23% were very concerned about a serious case, and 30% were somewhat concerned. Our sample overall is much younger than the PEW sample, which could account for some of the difference in attitudes toward the coronavirus and the vaccine.

Our key question about the vaccine is, “If the vaccine to prevent COVID-19 were available to you today, would you...” and response options were “Definitely get the vaccine”, “Probably get the vaccine”, “Probably not get the vaccine”, “Definitely not get the vaccine”, and “I have already gotten at least one dose of the vaccine”. 14.6% of respondents reported that they will “Definitely not get the vaccine”, and an additional 22.4% said they will “probably not get the vaccine”. When compared to national data, namely the PEW Research Center February survey, the percentage who say they will definitely not get the vaccine is very similar to the percentage from the PEW February survey (15%), but the percentage who say they will probably not get the vaccine (22.4%) is higher than in the PEW survey (15%). Combined, the percent who are against vaccination is higher among our sample than in the PEW sample of all adults. The combined share of respondents who either said they definitely will get a vaccine, probably will, or have already received a dose (63%) is very similar to the responses from the Black Americans who responded to the PEW survey in February (61%).

Our separate, key question relevant to attitudes toward the coronavirus and the vaccine is, “How much confidence, if any, do you have that the research and development process has produced a vaccine that is safe and effective?” Combined, about 40% of the respondents reported either “no confidence” or “not too much confidence”, which is about the same combined percentage opposed to vaccination. The modal response is “A fair amount of confidence”. Overall, this is a smaller percentage of respondents who have confidence in the science behind the vaccine compared to the PEW survey where only 25% of respondents indicated “no confidence at all” or “not too much confidence”.

We also chose to ask an additional question relevant to vaccine attitudes and behaviors: asking whether or not respondents were sharing a living space with a person who could be particularly vulnerable to the coronavirus. We asked “Are you living with or responsible for caregiving for an older adult or someone with a high risk of complications from COVID?” The four response options were “Yes, caregiving for 65+ person or high risk person”; “Yes, living with a person with a high risk of COVID complications”; “Yes, living with a person 65+ years old”; and “none of these apply to me”. As our respondents are mostly traditional aged college students, the modal response was “None of these apply to me”. However, 14% of respondents reported living with a person with a high risk of COVID complications, 4.5% reported living with someone over the age of 65, and 3.8% reported caregiving for a vulnerable person.

## 3. Results

**Table 3.** How much of a threat is the Coronavirus outbreak to your personal health?

	Frequency	Percent
A major threat	81	20.4
A minor threat	202	50.9
Not a threat	114	28.7
Total	397	100.0

**Table 4.** How concerned, if at all, are you that you might spread coronavirus to other people without knowing that you have it?

	Frequency	Percent
Not at all concerned	50	12.6
Not too concerned	96	24.2
Somewhat concerned	115	29.0
Very concerned	136	34.3
Total	397	100.0

**Table 5.** How concerned, if at all, are you that you will get the coronavirus and require hospitalization?

	Frequency	Percent
Not at all concerned	79	19.9
Not too concerned	155	39.0
Somewhat concerned	93	23.4
Very concerned	70	17.6
Total	397	100.0

**Table 6.** If the vaccine to prevent COVID-19 were available to you today, would you...

	Frequency	Percent
I have already gotten at least one dose of the vaccine	96	24.2
Definitely get the vaccine	67	16.9
Probably get the vaccine	87	21.9
Probably NOT get the vaccine	89	22.4
Definitely NOT get the vaccine	58	14.6
Total	397	100.0

**Table 7.** How much confidence, if any, do you have that the research and development process has produced a vaccine that is safe and effective?

	Frequency	Percent
A fair amount of confidence	170	42.8
A great deal of confidence	66	16.6
Not too much confidence	116	29.2
No confidence at all	45	11.3
Total	397	100.0

**Table 8.** Are you living with or responsible for caregiving for an older adult or someone with high risk of complications from COVID?

	Frequency	Percent
None of these apply to me	309	77.8
Yes, caregiving for 65+ person or high risk person	15	3.8
Yes, living with a person with a high risk of COVID complications (i.e. obese, diabetes, heart disease, etc)	55	13.9
Yes, living with a person 65+ years old	18	4.5
Total	397	100.0

### 3.1. Bivariate results

We recoded the vaccine question from five categories to just two categories: those who would “Probably not” or “Definitely not” get the vaccine and those who “Probably”, “Definitely”, or had already gotten the vaccine. This marks a clear distinction between those who plan to get the vaccine and those who were hesitant. Thus, our operationalization of vaccine hesitancy is those who were not planning to get the vaccine.

We expect that respondents' perceived risk would be related to vaccine hesitancy: generally, the more that people fear illness and death from the coronavirus, the more likely they are to plan to get vaccinated. We have derived four specific hypotheses from our literature review of vaccine hesitancy and from our Health Belief Model such that one's health could benefit or be susceptible to risks from receiving or not receiving the vaccine.

Hypothesis 1: Students who perceive the coronavirus to be a major threat to their personal health will be more willing to get vaccinated than students who perceive the coronavirus to only be a minor threat or no threat at all.

Hypothesis 2: Students who are living with others at risk for COVID complications will be more willing to get vaccinated than students who are not living with others at risk.

Hypothesis 3: Students who are concerned with spreading coronavirus to others will be more willing to get vaccinated than students who are not concerned with spreading coronavirus to others.

Hypothesis 4: Students who believe the vaccine is safe and effective will be more willing to get vaccinated than students who do not believe that the vaccine is safe and effective.

We performed bivariate chi-square tests of statistical significance for each hypothesis.

Hypothesis 1: Thirty-three percent of respondents who said that coronavirus is a major threat to their health do not plan to get the vaccine. In comparison, 37% of those who said that coronavirus is a minor threat to their health do not plan to get the vaccine. Finally, 39.5% of those who said that coronavirus is not a threat do not plan to get the vaccine. Overall, 37% of all respondents do not plan to get the vaccine. These differences are not statistically significant and the hypothesis is not supported. Students' perceptions of the coronavirus as a threat do not have an impact on vaccine hesitancy. If 37% of people never get the vaccine, then it is unlikely that we will ever achieve herd immunity from the coronavirus.

Hypothesis 2: We analyzed the crosstabulation data of living with a person at risk and vaccine hesitancy and found no statistical association. Of the respondents who are not living with someone at greater risk, 37% of them do not plan to get the vaccine. Of the respondents who are living with someone over the age of 65, 39% of them do not plan to get the vaccine. Of the respondents who are living with someone at risk due to comorbidities, 38% of them do not plan to get the vaccine. These small differences could be due to random error and there is a high probability we would produce results like those from a random sample even if there were no difference between groups. Therefore, there is not enough evidence to conclude that there is any difference in vaccine hesitancy based upon whether the students are living with a person who would be particularly vulnerable to COVID-19.

Hypothesis 3: We predicted that students who are concerned with spreading coronavirus to others would be more willing to be vaccinated (less hesitant) than students who are not concerned with spreading coronavirus to others. What we found is not statistically significant. About 33% of those who are “not too concerned” are vaccine hesitant, and 35% of those who are very concerned were vaccine hesitant. Those who were “not at all concerned” about spreading coronavirus did have a higher rate of vaccine hesitancy at 44%, however that difference is still not statistically significant.

Hypothesis 4: There is a large and statistically significant difference here. Students who have a “great deal of confidence” that the vaccine is safe and effective are almost all planning to get the vaccine (97%) with only 3% vaccine hesitant. In addition, 84% of students with a “fair amount of confidence” plan to get the vaccine (16% vaccine hesitant). In contrast, among students who responded that they have “not too much confidence” that the vaccine development resulted in a vaccine that is safe and effective, 64% are vaccine hesitant. Among students who had “no confidence at all”, 91% are vaccine hesitant. This idea of having confidence in the vaccine development process is by far the largest factor in explaining vaccine hesitancy.

### 3.2. Multivariate model

We present a binary logistic regression predicting vaccine hesitancy with the key independent variable being “Amount of confidence that the development process produces a vaccine that is safe and effective”. We have also included Survey Month, Age, Gender, Overall Health, Smartwatch Ownership, Frequency of Exercise, and Residential Student as control variables. Overall, this model can accurately predict vaccine hesitancy with 55% accuracy, with the key variable, Vaccine Confidence, by far the most influential variable. Those with no confidence in the vaccine are more than five times as likely to be vaccine hesitant compared to those with “not too much” confidence. Those with a fair amount of confidence or a great deal of confidence are much less likely to be vaccine hesitant. There are other variables in the model that have a statistically significant influence on vaccine hesitancy. Students who exercise on a daily basis are less likely to be vaccine hesitant compared to students who exercise less frequently. Male students are less likely to be vaccine hesitant compared to female students, and students who responded to the survey in April were less likely to be vaccine hesitant than students who responded in March. Respondents in June were also less likely to be vaccine hesitant than students who responded in March. Recall that in March, most students were not yet eligible for the vaccine.

**Table 9.** Binary Logistic regression predicting likelihood respondent is vaccine hesitant.

	B	S.E.	Wald	Sig.	Exp(B)
Age	0.03	0.02	1.40	0.237	1.03
Lives Off Campus (reference group on campus)	–0.25	0.32	0.61	0.435	0.78
Frequency of Exercise: Rarely/Never (ref group 2x per week)	–0.67	0.37	3.23	0.072	0.51
Frequency of Exercise: Daily* (ref group 2x per week)	–0.92	0.43	4.59	0.032	0.40
Gender1: Male* (ref group Female)	–0.97	0.49	3.95	0.047	0.38
Gender1: prefer not to say (ref group Female)	1.55	1.49	1.08	0.299	4.69
Smartwatch: NO (ref group has Smartwatch)	0.13	0.29	0.21	0.649	1.14
Overall Health: Average (ref group very good health)	0.09	0.36	0.07	0.797	1.10
Overall Health: (IDK/NA) (ref group very good health)	0.34	1.30	0.07	0.794	1.40
Overall Health: Excellent (ref group very good health)	0.40	0.61	0.43	0.513	1.49
Overall Health: Fair (ref group very good health)	–0.01	0.49	0.00	0.992	0.99
Overall Health: Poor (ref group very good health)	–0.21	0.89	0.05	0.818	0.81
Fair Amount of Confidence (Safe/Effective)*** (ref group Not Too Much Confidence)	–2.66	0.32	66.97	0.000	0.07
Great Deal of Confidence*** (ref group Not Too Much Confidence)	–4.31	0.80	29.31	0.000	0.01
No Confidence At All** (ref group Not Too Much Confidence)	1.66	0.58	8.32	0.004	5.27
Survey Month April* (ref Group March)	–1.09	0.48	5.22	0.022	0.33
Survey Month May (ref Group March)	–19.06	12515.78	0.00	0.999	0.00
Survey Month June** (ref Group March)	–1.04	0.41	6.62	0.010	0.35
Survey Month July (ref Group March)	–0.58	0.41	1.96	0.161	0.56
Constant	1.41	0.65	4.75	0.029	4.10
R-Squared: 0.551	

Note: Reference groups: “very good” Health; Female; “not too much confidence”; “twice week exercise”; “March survey month”.

### 3.3. Factors associated with vaccine reluctance

In addition to our key question about the vaccine, we also collected demographic data from the survey as well as data about exercise habits.

One key finding is that there are many students who don't have confidence in the science behind the vaccine and are concerned that the vaccine is not safe. Those students were much more likely to report that they were probably not or definitely not going to get the vaccine. In our survey there was an option for students to write in more detailed responses, and a few of those provide some insight into why some students do not trust the vaccine. We used content analysis to identify emergent themes and patterns in the responses from those who wrote in [37]. We first categorized all responses by topic, and then re-read those that were vaccine related to categorize those into sub-topics based on similar themes. We have included a few representative quotes to illustrate the most common themes identified in the students' responses. All names are pseudonyms to protect the identity of research subjects. The three themes that were most prevalent among the vaccine hesitant respondents were 1) fear of negative side effects, 2) skepticism about the effectiveness, and 3) a sense of the vaccine being “unnatural”.

There are some who are fearful of the side effects of the covid vaccine. Here are a couple examples of students' statements that echo that sentiment:

*“I personally would not take the vaccine because I have heard people getting sick after receiving it. I have never gotten the flu shot and will not be getting the covid vaccine.” — [Tori]*

*“My cousin recently got the vaccine and she started to get headaches, body aches, and chills. This made me skeptical about taking the vaccine.” — [Lavanna]*

Both Lavanna and Tori are young traditional aged underclass students. Lavanna and Tori both took the survey in late March of 2021. Both indicate that the coronavirus is only a ‘minor’ threat to their personal health, and both indicated that they are not concerned that a COVID case would result in hospitalization. Each student indicated that they were not living with anyone who would be at risk for severe complications. Lavanna and Tori have close experience with friends or family experiencing negative side effects, and these experiences have scared them from taking the vaccine themselves.

Another student also specifically mentioned negative side effects as a reason for not getting the COVID vaccine:

*“I have been a nurse for more than 20 years and I do not feel that the vaccine has been studied enough for me to take it and possibly negatively impact my health as I am personally aware of complications that have developed as a result of the vaccine. I still wear my mask in public and limit my interactions”* — *Charity*

This student [Charity] is in their late 40s and completed the survey in early July. Charity indicated they don't have confidence that the vaccine is safe and effective and they consider themselves to be in good health currently, and also that COVID is not a great threat to their personal health. They don't think it is likely that they'll be hospitalized if they do happen to get COVID. Despite their own perceived lack of risk from COVID, they also answered that they were living with another person who is at risk of severe complications from COVID if they were to get it due to comorbidities. However, that person's health does not seem to have factored into their own thoughts about getting vaccinated. For Charity and the other two students mentioned above, from their perspective the vaccine is more of a threat to their health than the coronavirus.

A separate group of qualitative responses focused on skepticism of the effectiveness of the vaccine:

*“Personally I don't trust the vaccine. I need to see more results from those that have taken it already. I just see it like this: how did they come up with a vaccine for an infection/sickness that we literally knew nothing about in less than nine months, but have been studying and trying to find a cure/vaccine for HIV/AIDS for 20+ years with all the information we know about that. Doesn't make sense to me.” — [Kenya]*

Kenya took the survey in late March, before she and her peers would've been eligible for the vaccine. Her comment indicating “I need to see more results from those who have taken it” is rational behavior for anyone who does not have complete faith in the medical establishment. She further elaborates that she is suspicious of the rapid development of the vaccine compared to the lack of a cure for HIV/AIDS despite the medical establishment working on that for decades.

Effectiveness: *“I don't feel confident knowing that the lead of the vaccine research has stated that the vaccine will not protect against transmission or contraction and the level of adverse events related to the vaccine when rates of mortality do not even surpass that of the flu. So, I'm good” — [Alexis]*

*“My family has taken the vaccine but I don't think it is all the way effective yet because neither shot is in the 90%” — [Daisha]*

In response to the survey question about whether or not COVID is a personal threat to them, both Alexis and Daisha responded that it is not much of a threat, and they are also not too concerned about spreading it to others, and not too concerned about possible hospitalization if they were to become infected. Despite that, Daisha describes herself as overweight and describes her own health as fair. Additionally, she reports that she is living with a person who is at risk of COVID complications. Alexis also reports that she is currently caregiving for a person over the age of 65. So, according to their own descriptions, they are at risk of potentially endangering other people with whom they live, but they definitely do not plan on getting the vaccine. Alexis, however, is absolutely correct that the vaccine has not been proven to protect against transmission or contraction. This is perhaps a failure in the way that the scientific community has communicated about the role of the vaccine in ending the pandemic. The vaccine provides protection against severe illness, hospitalization, and death, however, it does not prevent transmission. Daisha took the survey in late March and Alexis took the survey in late June. Alexis is incorrect in her statement that the rates of mortality do not surpass that of the flu, as coronavirus is substantially more likely to lead to mortality than the flu.

Finally, there are those that are opposed to the vaccine due to a sense of it being an “unnatural” element to their bodies that they do not trust. Here are two quotes from students who have expressed this “herbalist” mindset:

*“For me I am a herbalist it is actually what both of my grandmothers did. So my health is very important to me I am vegan and raw vegan on most days no artificial anything. I make sure to workout every day twice a day and take my immunity building teas and herbal blends. I have a African wholistic perspective to vaccinations” — [Patricia]*

*“I feel like they just came up with the vaccine they are a lot of natural herbs and home remedies that can save your life and help with preventive measure. I come from a Island family and all we do is natural medicines” — [Ebony]*

Both Ebony and Patricia are in their late 20s. Neither of them has confidence that the vaccine is safe and effective. Patricia responded that she is probably not getting the vaccine, while Ebony responded that she is definitely not getting the vaccine. Ebony is living with a person with a high risk of COVID complications, and Patricia is living with someone over the age of 65. Patricia responded that COVID is “not a threat” to her personally, is not concerned with the possibility of getting COVID and requiring hospitalization, and is not concerned that she could spread COVID to others. In contrast to Patricia's lack of concern about the virus, Ebony considers COVID to be a major threat, and is very concerned about the possibility of requiring hospitalization and very concerned about the possibility of spreading COVID to others. Despite this, she believes that home remedies and preventive measures are the best approach to dealing with COVID and will not be getting vaccinated. Patricia responded to the survey in early April, before she was eligible to receive the vaccine. Ebony responded in early July, when the vaccine was widely available to all adults.

## 4. Discussion

We have presented data from students at an HBCU to assess attitudes and behaviors toward COVID-19 vaccines. Results from this study contribute importantly to our understanding of vaccine perceptions and behaviors in a high-priority vaccination population — HBCU students. Our sample of mostly young, African American college students provide both quantitative and qualitative insights about their concerns, adding to the growing body of literature examining uptake and skepticism according to age and racial/ethnic background. Additionally, our survey included several key questions about health behaviors more generally, which allows us to examine the role of factors such as frequency of exercise.

All data was collected after the rollout of the vaccines in early 2021. The results indicate that as of July 2021, vaccine uptake in our sample of students (39%) was far below the national target of 70%. We found a high amount of vaccine hesitancy, with 37% of students in the sample stating that they probably or definitely would not get the vaccine. The single biggest predictor of vaccine hesitancy was perceptions that the vaccine was not safe. The qualitative responses among those who were vaccine hesitant indicated that vaccine hesitancy related to fear of side effects, as well as distrust of vaccine efficacy and the development process.

Overall, the Health Belief Model provides a worthy framework for understanding vaccine uptake and skepticism, especially as it pertains to perceived susceptibility and severity as a predictor of health behaviors. Indeed, our sample of younger adults expressed less perceived threat. Additionally, our multivariate model revealed statistically significant associations with gender and frequency of exercise, with women and those who exercise frequently more likely to be hesitant to get vaccinated. Both of these groups might be more risk tolerant due to perceived invulnerability, however there is a need for future research into these factors.

A discussion of several key implications follows from these findings. First, the context of our sample population is important to consider. African Americans in particular have legitimate reasons to question the safety of medical interventions. Structural racism in the U.S. medical system, negative interactions with medical providers, and a history of medical experimentation on African Americans without their consent have created an environment of distrust. Dovetailing with the skepticism found in our sample is the disproportionate impact COVID-19 has had on the African American community. Because of these dual concerns, efforts to earn the trust of African Americans are urgently needed. Trust for America's Health (TFAH) has called for equitable vaccine distribution and access, including funding to support the administration of shots through community-based organizations [38].

Second, vaccine hesitancy has been shown to be surmountable. One recent survey reported that 34% of respondents who said between January and March 2021 that they were unlikely to get vaccinated later received at least one dose by July 2021 [39]. Because safety concerns were strongly associated with vaccine skepticism in our sample, it is important to unpack the bases of their concerns. Echoing the findings of Batelaan [29], we argue that writing off this group of vaccine skeptical students as “anti-vaccination” is incorrect. Indeed, the safety concerns expressed in the qualitative responses of many participants lend themselves to authentic, factual conversations that can respond to and assuage legitimate fears expressed by this population.

Third, HBCU students provide an important opportunity for much needed improvement in vaccine uptake within our overall population. Indeed, African Americans and other marginalized groups are essential components of our societal goals for herd immunity. Yet, the continued devaluation of Black life in U.S. society has led to a paradox. Namely, viral mutation persists in unvaccinated populations where COVID-19 continues to spread [40]. If the dominant institutions of our society continue to ignore the African American population's safety concerns and need for vaccination, we will continue to see the uncontrolled spread of COVID-19 throughout the United States. Hence, our sample population represents a key component of the United States' overall public health response. However, our study is limited by several key factors: because a large number of students sampled were attending school in the summer, they might not necessarily be representative of the college's overall population. Furthermore, with the setting being an urban HBCU, it might not be generalizable to other HBCUs in more traditional “college towns.”

Because of the disproportionately negative impact of COVID-19 on African Americans, there is a tremendous need for further research and policy measures to increase vaccination and reduce harm of the disease in this population. Longitudinal data is needed to determine whether and how vaccine skeptical individuals change their vaccination perceptions and behaviors over time, as new information comes to light and the pandemic evolves. What sorts of interventions are most effective in changing these perceptions and behaviors? As more institutions begin to mandate COVID-19 vaccination, research is needed to explore how such mandates will impact this population. In the context of rapidly changing public health guidance, rules, and cultural norms around this disease, a better understanding of HBCU students navigating the pandemic stands to benefit not only community health, but also educational and wellness outcomes.

## 5. Conclusions

Racial disparities in COVID-19 infections, deaths, and vaccinations serve as a stark reminder of the urgent need to better understand the factors that could lead to mitigation of the virus. Fear about the safety of the vaccine among minority populations in particular must be unpacked in order to address valid concerns and overcome hesitancy. This study provides key insights into the contours of those fears.

The historical and structural roots of the mistrust identified in this study run deep. But as others have found, community-based efforts to earn the trust of high-priority vaccination populations can and do pay dividends for the health and wellbeing of all.

As scientists and researchers grapple with worldwide public hesitation in relation to the COVID-19 vaccine, many are seeking to respond to the question, “How do we address vaccine hesitance?” While there is no clear answer to this question, our findings point to several considerations for encouraging HBCU students to get vaccinated. Among the appropriate responses to addressing the vaccine hesitance of HBCU students is to counter misinformation and fears with culturally responsive public health educational messaging. The students in our study often expressed concern about potential reactions to the COVID-19 vaccine. With that, organizing events to educate individuals about potential effects of the vaccine, rather than simply encouraging vaccination, could help HBCU students to feel more comfortable and informed. It might also be helpful to organize seminars in which HBCU students could talk with multiple entities, including health professionals, peers who may have personally been diagnosed with COVID-19, and peers who have received the COVID-19 vaccine.

In addition to addressing the immediate vaccine hesitance among HBCU students, a broader conversation around countering the historical distrust of medical interventions is needed in the Black community. COVID-19 exposed deep-seated concerns about trusting medical professionals and politicians. While these concerns are certainly not new, when paired with an immediate need to encourage vaccination, historical distrust likely worsened disparities contributing to the disproportionate rate of deaths from COVID-19 within the Black community. There is an urgent need to address vaccine hesitance within minority communities and address these disparities. HBCUs are prime spaces for engaging Black students, and HBCUs should be venues in which diverse groups seek to educate students on the importance of understanding and overcoming barriers to racial health equity.
